# Bioactive Potential of 2-Methoxy-4-vinylphenol and Benzofuran from *Brassica oleracea* L. var. *capitate f, rubra* (Red Cabbage) on Oxidative and Microbiological Stability of Beef Meat

**DOI:** 10.3390/foods9050568

**Published:** 2020-05-04

**Authors:** Momna Rubab, Ramachandran Chelliah, Kandasamy Saravanakumar, Kaliyan Barathikannan, Shuai Wei, Jong-Rae Kim, Daesang Yoo, Myeong-Hyeon Wang, Deog-Hwan Oh

**Affiliations:** 1 Department of Food Science and Biotechnology, College of Agriculture and Life Sciences, Kangwon National University, Chuncheon 200-701, Korea; rubab.momna@gmail.com (M.R.); ramachandran865@kangwon.ac.kr (R.C.); bkannanbio@gmail.com (K.B.); chief1111@daum.net (J.-R.K.); daesangy@naver.com (D.Y.); 2 Department of Medical Biotechnology, College of Biomedical Sciences, Kangwon National University, Chuncheon 200-701, Korea; saravana732@gmail.com (K.S.); mhwang@kangwon.ac.kr (M.-H.W.); 3 College of Food Science and Technology, Guangdong Ocean University, Zhanjiang 524088, China; weis@gdou.edu.cn; 4 Hanmi Natural Nutrition Co., LTD 44-20, Tongil-ro 1888 beon-gil, Munsan, Paju, Gyeonggi 10808, Korea; 5 H-FOOD, 108-66, 390 gil, Jingun Oh Nam-Ro, Nam Yang, Ju-Shi, Gyung Gi-Do 12041, Korea

**Keywords:** red cabbage (RC), phytochemicals, antimicrobial, *C. elegans*, cytotoxicity, beef preservation

## Abstract

In the future, plant based phytochemicals will be considered as efficient replacement sources of chemical preservatives, to act as potential bio-preservatives. We investigated the antibacterial and antioxidant activity of red cabbage (RC) extracts using different solvents. Among all extracts, chloroform extract exhibited strong antimicrobial and antioxidant activities. Hence, the phytochemical constitutions of the RC chloroform extract was examined by GC-MS analysis, and further, based on molecular docking analysis, revealed 2-Methoxy-4-vinylphenol and benzofuran as two major compounds found to be possessing higher degrees of interaction with DNA gyrase (4PLB; −8.63 Kcal.mol^−1^) and lipoprotein (LpxC−8.229 Kcal.mol^−1^), respectively, of the bacterial cell wall, which leads to higher antimicrobial efficacy. Further, it was confirmed with that the in vivo *Caenorhabditis elegans* model (but no cytotoxic effect) was exhibited in the MCF-7 cell line. Thus, we investigated the influence of this extract on the shelf life of meat under refrigeration storage. The physicochemical properties were observed periodically, and microbial analysis was conducted. The shelf life of the beef was enhanced (up to eight days) in terms of microbial and physiochemical properties, at 4 ± 2 °C when compared to control. We concluded that chloroform extract of RC has potential as a natural preservative in the meat processing industry.

## 1. Introduction

In the 21th century, for consumers in both European and Asian countries, there has been increasing demand for meat products based on their beneficial health effects. However, they are vulnerable to microbial spoilage due to the high moisture content and enriched nutrition profile [1]. The increasing need for meat products has led to the development of preservation technology; this prevents food spoilage and improves public health research [2]. Dysentery pathogens, including the toxin-producing Enterobacteriaceae group, are mainly responsible for causing meat spoilage [3]. To ensure food safety, a variety of synthetic additives and chemical preservatives are extensively applied in the meat industry [4]. The positive aspects of chemical preservatives executes efficient antimicrobial effects against foodborne pathogens and predominantly extends the storage life of the meat products; however, the negative side effects of chemical preservative leads to numerous health problems [4]. Nowadays, consumers question the safety of chemical and synthetic preservatives in food, as well as the health effects. Therefore, considerable efforts have been made to restrict the use of chemical preservatives in food applications, which are impregnable for consumer health [5]. This has opened a new dimension for the application of bio-preservatives based on natural plant-derived phytochemical preservatives towards antimicrobial and antioxidant activities in food. The majority of laboratories across the globe are involved in systematic screening of plant species for revealing new bioactive compounds. Therefore, there is a need for scientific affirmation of bioactive compounds [4]. Numerous edible and herbal plants were screened for antimicrobial activity in recent times [5]. Natural products can be beneficial for inhibition of both microbial and oxidation-induced food spoilage since they exhibit both antimicrobial and antioxidant activities. Plants were considered ample sources of the wide range of secondary metabolites responsible for antimicrobial potential, such as tannins, terpenoids, alkaloids, and flavonoids [5].

*Brassica oleracea* L. var. *capitate* *f*, *rubra*, commonly known as red cabbage (RC), and belonging to the family Brassicaceae, is one of the most important vegetables grown globally. The lack of studies regarding in vitro antioxidant and antimicrobial potential of RC has prompted the current research. Therefore, the aim of this work was to investigate the antimicrobial and antioxidant potential of RC extracts using different solvents in vitro. Based on in vitro results, extract with strong antimicrobial and antioxidant ability was subject to cytotoxic evaluation, to assess its safety index and GC-MS analysis, coupled with molecular docking, to identify the potential bioactive lead compound(s) responsible for its biological activities. To investigate the potential of using the active RC extract as a natural preservative, we selected beef meat as a model in raw meat systems, and studied shelf life extension properties for 16 days at 4 °C.

## 2. Materials and Methods

### 2.1. Extraction Procedure

Fresh cabbage heads were randomly procured from the local market in South Korea and then transported to the laboratory. The stem of cabbage was removed, and the leaves were washed and dried at 75 °C oven temperature, and pulverized into a fine powder using a stainless steel blender. The 2 g of ground powder was subject to extraction in each bottle, having different solvents (toluene, diethyl ether, chloroform, dichloromethane, ethanol, methanol, and distilled water; 50 mL for each) at room temperature (RT) for 24 h with continuous stirring at 150 rpm. Extracts were then evaporated using a vacuum rotary evaporator. The samples were collected by using dimethyl sulfoxide (DMSO) for non-polar extracts (chloroform, dichloromethane, diethyl ether, and toluene) and distilled water for polar extracts (ethanol, methanol and distilled water), by stirring for 3 h using a magnetic stirrer following evaporation. After that, extracts were separated from the residue through a centrifuge for 30 min with 4000 rpm and stored at 4 °C until use.

### 2.2. Total Phenol (TP), Total Flavonoid (TF) Content, and Antioxidant Activity of Red Cabbage

#### 2.2.1. TP and TF Content

The total phenol (TP) and total flavonoid (TF) were measured spectrophotochemically using the Folin–Ciocalteu (F–C) method of Rubab et al. [6]. Briefly, 2.5 mL of 10% Folin–Ciocalteu reagent was added to the 0.5 mL of the extract and a standard solution of gallic acid (GA). The solutions were incubated at RT for 3 min, after which 2.5 mL of 7.5% NaHCO_3_ was added and incubated again for 90 min in the dark. The absorbance was observed at 765 nm and TP content expressed as mg of GA equivalent per g dried RC. For TF content, 100 µL of 2% aluminum chloride was added to 250 µL of the extract and a standard of quercetin. The solutions were incubated at RT for 1 h and absorbance was observed at 415 nm. The TF content was expressed as mg of quercetin equivalent per g dried RC (mg QE g^−1^).

#### 2.2.2. Antioxidant Activity Assays

The radical scavenging activities of the cabbage extracts were quantified using 2,2’-azino-bis(3-ethylbenzothiazoline-6-sulfonic acid (ABTS) and 1-diphenyl-2-picrylhydrazyl (DPPH) radical as a reagent, by the method of Rubab et al. [6]. The absorbance was observed at 517 nm in DPPH assay. The DPPH radical scavenging activity was described as inhibition percentage and scavenging capability was figured as follows;

(1) $$\mathrm{DPPH}\;\mathrm{scavenging}\;\mathrm{effect}\;(\%\;\mathrm{inhibition})=\frac{A_{0}-A_{1}}{A_{0}}\;\times \;100$$

where A_0_ is the absorbance of the control (only DPPH without sample) and A_1_ is the absorbance with the sample and the reference.

In ABTS assay, absorbance was observed at 734 nm. The ABTS scavenging activity was exhibited as inhibition percentage and scavenging capability was figured as follows:

ABTS radical scavenging activity (%) = (A control − A extracts)/A control × 100(2)

### 2.3. GC-MS Analysis

GC-MS analysis was conducted by using an Agilent 7890 A, 5975C system equipped with an Agilent capillary column HP DB-5 (30 × 0.25 mm; film thickness 0.25 μm) method of Bakari et al. [7], with some modifications. The temperature program was set from 50 to 280 °C at a rate of 5 °C.min^−1^, with the split ratio of 10:1. GC-MS was operated in a continual flow mode 5.0 mL.min^−1^. The injection volume was 2 µL and hydrogen was used as carrier gas. The identified compounds were detected by comparing the mass spectra with those of valid samples acquired by MS library.

### 2.4. Antimicrobial Activity

#### 2.4.1. Microorganisms

A total of 7 bacterial strains and 4 fungal strains were used to investigate the antimicrobial potential of RC extracts (Table 1). All bacterial strains were subculture in nutrient broth (NB) and fungal strains in De Man, Rogosa, and Sharpe (MRS) broth at 37 °C and 30 °C for 24 h, respectively.

#### 2.4.2. Antimicrobial Assay

Disc diffusion method was used to screen the antimicrobial activities of different RC extracts. Gentamicin (30 µg.disc^−1^) and sodium metabisulfite was employed as positive control and DMSO as negative control. Briefly, 100 µL of fresh bacterial or fungi culture was pipetted in the center of agar plates (Muller–Hinton agar (MHA) for bacterial and De Man, Rogosa, and Sharpe (MRS) for fungal strains). Then, Whatman filter paper discs (8 mm) were aseptically placed on the surface of agar with 100 µL of RC extracts. The MHA and MRS agar plates were incubated at 37 °C and 30 °C for 24 h, respectively, and examined for inhibition zones. Antimicrobial activity was determined by measuring zone of inhibition (including the disc diameter). Antimicrobial test for all microorganisms were conducted in triplicate (3 petri dishes) and the results described, herein, are mean values of 3 petri dishes. Only samples with strong antimicrobial activity were used to evaluate the minimum inhibitory concentration.

#### 2.4.3. Determination of Minimum Inhibitory Concentration (MIC)

The MIC of the selected RC extract was quantified according to the method of Bussmann et al. [8] using a 96-well microplate. The MIC was performed for *Staphylococcus aureus* ATCC 13,150 and *Escherichia coli* ATCC 35150. Briefly, 0.5 mL of the sterilized NB broth filled into the 7 wells of each row. Sequentially, an additional 0.5 mL of a mixture of culture medium (10^6^ CFU.mL^−1^) and RC extract serially diluted to make a concentration range from 3.30–33 mg.mL^−1^ and well 1 served as growth control. The microplate was then incubated at 37 °C for 24 h and the resulting turbidity was observed using spectrophotometer at the optical density (OD_600_ nm). The MIC was defined as the lowest concentration of the RC chloroform extract, which exhibited clear fluid with no evolvement of turbidity.

#### 2.4.4. Thermal Stability

The thermal stability of red cabbage chloroform (RCC) extract was evaluated by following the methodology proposed by Rubab et al. [6]. Briefly, the extract was heated at 95 °C for 5, 45, and 90 min. Thereafter, the antimicrobial potential was estimated by the disc diffusion method as described in Section 2.4.2.

### 2.5. Docking Method

An in silico docking study was applied to investigate the interactions between the compounds, from the cabbage extracts and two target bacterial proteins (lipoprotein; LpxC and bacterial type II topoisomerase inhibitor; NBTI). The crystal structure of bacterial targets proteins such as LpxC (PDB ID: 3U1Y) UDP-3-*O*-(*R*-3-hydroxymyristoyl)-*N*-acetylglucosamine deacetylases, and novel bacterial topoisomerases inhibitors (PDB ID: 4PLB) [9] were obtained from the protein data bank (https://www.rcsb.org/). The compound (ligands) structure was prepared using the ACD/ChemSketch based on the canonical SMILES procured from NCBI (https://www.ncbi.nlm.nih.gov/pccompound). Receptor and ligands were pretreated using the standard method as described earlier [10]. Moreover, the interacts and docking energy were calculated using the Argus Lab 4.0.1 (Mark Thompson and Planaria Software LLC) and BIOVIA Discovery Studio 2016 (Accelrys Software Inc., San Diego, CA, USA). Further, the results of molecular docking were verified by in vitro antimicrobial experiments for 5-Methylfuran-2-carbaldehyde (hydroquinone; CAS number: 123-31-9), 2-methoxy-4-vinylphenol (CAS number: 7786-61-0), 2-Purinol (hypoxanthine; CAS number: 68-94-0), benzofuran (2,3-benzofuran; CAS number: 271-89-6), 4H-Pyran-4-one (CAS number: 108-97-4), and 2-Furancarboxaldehyde (furfural; CAS number: 98-01-1).

### 2.6. Cytotoxicity Assay

The cytotoxicity of the different extracts on breast cancer cell-line (MCF-7) cells was measured using MTT assay by following the previously reported methodology [6].

### 2.7. In Vivo Analysis of Cytotoxicity

#### 2.7.1. *C. elegans* Culture Conditions

The wild-type (N2) *Caenorhabditis elegans* strains were obtained from the *Caenorhabditis* Genetic Center (CGC; MN). Prior to further experiments, worms were cultured and maintained on standard Nematode Growth Media (NGM) medium at 20 °C with living OP50 *E. coli* as food source.

#### 2.7.2. Chemotaxis Assay

The chemotaxis assay was performed by following the methodology of Chelliah et al. [11], with some modifications. Briefly, 10 µL of OP50 were seeded with worms at the L4 stage. The 50 young adult stage worms were transferred to a 5 cm plate (in center), and movement of worms were monitored using a two-quadrant system. The movement of worms were monitored and counted after 90 min of incubation at 25 °C. Chemotaxis assay was conducted in triplicate; chemotaxis index (CI) was quantified according to Chelliah et al. [11] and expressed in CI%, referring to the number of worms in the extract by the total number of worms used.

#### 2.7.3. Colonization Assay (In Vivo Antimicrobial Activity)

Colonization assay was conducted according to the methodology of Chelliah et al. [11] with some modifications. Briefly, extract plus OP50 were seeded on NGM plates, having worms, and incubated for 9 days, and the total number of worms counted first at the third day of incubation, and then with an interval of 2 days until the end of incubation period. At each tested day, 5 worms were picked and washed in 5 µL drops of Triton x-100 (1%) for paralysis, and to hinder pharyngeal pumping and expulsion. The washed worms were transferred to an Eppendorf tube, having 50 µL of Triton x-100 (1%), and disrupted mechanically using a pestle and mortar. Worm lysates were then serially diluted in phosphate buffered saline, and incubated on Eosin Methylene Blue agar, overnight, at 37 °C. *E. coli* colonies were counted and enumerated the number of bacteria/nematodes.

### 2.8. Application of RCC Extract on Raw Beef Meat

#### 2.8.1. Beef Sample Preparation and Storage Conditions

Fresh boneless beef was procured from a local supermarket, stored immediately in an insulated polystyrene box, and kept at 4 °C for further analysis. Beef fillets were then aseptically cut to 50 g piece and divided into three categories. Each category received 5 pieces of beef for subsequent microbial and quality analysis. The samples were assigned to one of three treatments: C: control (no addition), RCC-A: 1.5%, and RCC-B: 2% red cabbage chloroform (RCC) extract. The treatment was given for 20 min, the respective extract, and then drained for 1 h in a safety bench. The treated samples and controls were separately packaged in low-density polyethylene bags (Whirl-Pak, Nasco, Fort Atkinson, WI, USA) and stored at 4 °C for up to 16 days. The beef samples were investigated with 4 days of interval for microbial, pH, instrumental color attributes moisture, texture, and thiobarbituric acid reactive substances (TBARS); the above-described experiment was performed in triplicate on days 0, 4, 8, 12, and 16.

#### 2.8.2. Microbial Analysis

The 10 g of meat samples were homogenized with 90 mL of sterilized 0.1% buffered peptone water (BPW) in a sterile stomacher bag for 2.5 min in a bag mixer (BagMixer, MonotaRO Co., Ltd., Japan). The sample dilutions (0.1 mL) of appropriate dilution in 0.1% BPW of beef homogenate were plated on the surface of different selective agars. The plate count agar (PCA) was used for total viable count (TVC), and total psychrotrophic bacteria count (TPC), and incubated at 37 °C for 48 h and at 7 °C for 7 to 10 days, respectively. The Dichloran Rose Bengal Chloramphenicol (DRBC) agar was used for yeast and molds, and incubated at 25 °C for 3 to 5 days. The microbiological counts were transformed to log_10_ CFU.g^−1^ of beef meat.

#### 2.8.3. Physiochemical Analysis

##### pH Analysis

The pH value was evaluated according to [12]. Briefly, each sample (10 g) was homogenized with 90 mL distilled water and homogenized for 1 min in homogenizer (Ultra-Turraz, T25-S1, IKA, Staufen, Germany). The pH was measured using a pH meter (Mettler Toledo SB 8001; Shanghai Mettler Ltd., China).

##### Color Measurements

Color was measured using a MiniScan XE Plus Hunter meter (HunterLab Associates Inc., Reston, VA, USA) right on the beef surface. The color was expressed as L* (lightness), a* (redness), and b* (yellowness).

##### Texture Profile Analysis (TPA)

The TPA of the beef sample was conducted using an instrumental texture analyzer (FRTS-50N; IMADA CO., LTD, Japan) by following the methodology of Barekat and Soltanizadeh [13], with slight modifications. The following texture parameters were evaluated: hardness, springiness, gumminess, chewiness, and cohesiveness, by compressing 20% deformation forces, using a compression load (7 kg) with a probe diameter of 30 mm on meat surface.

##### Moisture Analysis

The Association of Official Analytical Chemists (AOAC) method was used to determine the moisture content of beef sample with and without treatment (925.10, [14]). Approximately 5 g of the beef sample from each treatment with 4 day intervals was weighed aseptically, then the solid content was examined after complete dehydration of the beef sample, using for 20 min at 140 °C in moisture analyzer (WBA-110M 0.01–110 g; WITEG LABORTECHNIK GMBH, Germany). The percentage decline in weight was demonstrated as moisture content, the experiment was performed in triplicate, and the average was considered as the moisture content of the product.

##### Thiobarbituric Acid Reactive Substances (TBARS)

The TBARS values were determined to evaluate the lipid stability of the beef using a modified method based on Du and Ahn [15]. Concisely, 5 g of beef sample was transferred to a polystyrene bag contained 15 mL of deionized distilled water (DW) and homogenized for 2 min. Then 1 mL of beef homogenate was fetched to a test tube having 2 mL of 15 mM thiobarbituric acid (TBA) and trichloroacetic acid (15%; TCA). The 50 µL of 7.2% (w/v) solution of butylated hydroxytoluene (BHT) was added to each test tube and was further vortexed for few seconds. The samples were then placed in the boiling water bath for 15 min to generate color, cooled to room temperature for 10 min, and the absorbance was observed at 531 nm by employing spectrophotometer against deionized water blank. The TBARS values were derived from a calibration curve (TEP) and demonstrated as µmol.kg^−1^ of the beef sample.

### 2.9. Statistical Analysis

All experiments were conducted in triplicate and expressed as mean ± standard deviation (SD). The data were analyzed using SAS program (SAS Institute Inc., USA). Comparison between the groups or treatments were conducted using one-way analysis of variance (ANOVA) and Tukey’s test. Differences at *p* < 0.05 were considered significant.

## 3. Results and Discussion

### 3.1. Antimicrobial Activity

Phytochemical based antimicrobial compounds from plant sources have been renowned to possess broad-spectrum antimicrobial properties [1]. Particularly, two major groups of polyphenols, phenolic compounds and flavonoid compounds, were reported to hinder a wide range of pathogenic microorganisms [1,5]. The mechanism of the plant based antimicrobial action on microorganisms is attributed to the inhibition of synthesis of nucleic acid, immobilization of cytoplasmic membrane function, and non-specific reactions in energy metabolism; however, accurate mechanisms remain to be determined [16]. Previously, a number of studies have been reported, phytochemical based antimicrobial compounds; however, red cabbage extract (used in this study) has not yet been evaluated for its effectiveness towards foodborne pathogens and its utilization in a raw meat system, in terms of food safety.

In this study, the antibacterial and antifungal activities of RC from different solvent extracts were investigated towards fungal and bacterial pathogens using the disc diffusion method (Table 1). All extracts could hinder the growth of the microorganisms to different extents; however, RCC extract exhibited a more effective hindrance, with the highest zone of inhibition for *S. aureus* ATCC 13,150, and *E. coli* ATCC 35,150, with the diameter of 14.00 ± 0.04 and 13.00 ± 0.02 mm, respectively. Overall, all tested fungal strains were more sensitive to RCC extract when compared to other extracts. The antimicrobial activity exhibited by RCC extract was equivalent to that of the standard antibiotic gentamicin and sodium metabisulfite; however, no inhibitory activity was perceived towards the negative control for any of the test microorganisms (data not shown here). Gentamicin was inhibitorier to most of the bacterial strains tested. 

Further, thermostability of the chloroform extract was evaluated using the disc diffusion method. All heat treatments exhibited the antimicrobial activity towards the organisms tested in this study, so we can say that the compound liable for antimicrobial activity may not be protein in nature (Table 2).

Previously, a number of studies have reported the RC extract of plants possessing antimicrobial activity [6,17]. These results showed that the extracting solvents have a defined impact on bioactive postulates. Generally, the extracts exhibiting a big zone of an inhibition zone, in terms of diameter, with low minimum inhibitory concentration, can be acknowledged as a more effective bioactive agent than that of a small zone of inhibition and high MIC [18]. A micro-dilution assay was conducted to evaluate the susceptibility of the *S. aureus* and *E. coli* against RCC extract and the results are expressed as MICs. The RCC displayed the MIC values of 16.5 mg.mL^−1^ against *S. aureus* and *E. coli*. In the current study, RCC extract was the most effective extract that presented broad-range antimicrobial ability, hinder growth, of all microorganisms tested in this study.

### 3.2. Phenolic and Flavonoid Content

Preliminary phytochemical screening of different solvent extracts of red cabbage revealed the presence of flavonoids, alkaloids, saponins, and tannins (Table S1). Phenolic and flavonoid compounds are substantial plant components due to their biological activities and scavenging ability because of their hydroxyl group [19]. The crude RCC extract exhibited the highest TP content (85.48 ± 1.60 mg GAE.g^−1^), followed by dichloromethane (79.52 ± 2.32 mg GAE.g^−1^), ethyl ether (78.51 ± 1.96 mg GAE.g^−1^), toluene (81.25 ± 1.65 mg GAE.g^−1^), ethanol (78.63 ± 2.40 mg GAE.g^−1^), and methanol (78.75 ± 1.80 mg GAE.g^−1^) extracts, with a significant differences between them (*p* < 0.05). Phenols are a group of renowned natural antioxidants, comprising hydrolyzable and non-hydrolyzable tannins, flavonoids, lignins, and terpenoids [20,21]. Among all extracts, the highest level of the TFC was recorded for chloroform (28.70 ± 3.12 mg QE.g^−1^), followed by dichloromethane (26.50 ± 2.40 mg QE.g^−1^), ethyl ether (23.01 ± 1.70 mg QE.g^−1^), toluene (21.85 ± 1.90 mg QE.g^−1^), ethanol (18.20 ± 2.60 mg QE.g^−1^), and methanol (15.20 ± 2.45 mg QE.g^−1^) extracts. The variation in the concentration of phenolic compounds indicated in literature depends on the choice of solvent. The results indicated that the solvent extract significantly impacted the extraction capacity of the phenolic compounds, in terms of solubility of the polyphenols in the solvent employed for the extraction process [22]. Our results are consistent with previous studies, exhibiting that chloroform is one of the best solvents to extract a wide range of phenolic components from plants.

### 3.3. Antioxidant Activity

**Figure 1:**
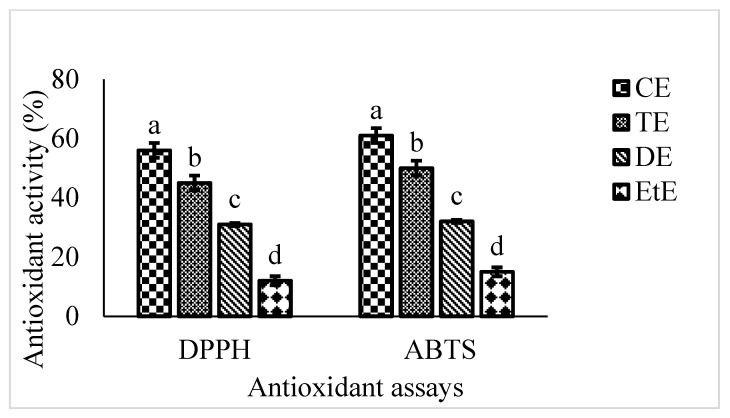
Antioxidant activity of different extracts of RC. CE: Chloroform extract, TE: Toluene extract, DE: Dichloromethane extract, and ETE: Ethyl ether extract. Letters a–d indicates the significance difference among different extracts of RC.

Two extensively used antioxidant assays (ABTS and DPPH assay) were employed to evaluate the antioxidant efficiency. Different extracts were quantified by their scavenge on DPPH and ABTS radicals, and ascorbic acid utilized as positive control (Fig.0ure 1). All tested extracts exhibited the DPPH radical activities, ranging from 12%–56%, and ABTS radical activities, ranging from 15%–61%. In general, the chloroform fraction exhibited the strongest antioxidant activity and, subsequently, toluene and dichloromethane. As exhibited in Figure 1, DPPH and ABTS radical scavenging values were found to be in the order of chloroform extract < toluene extract < dichloromethane extract < ethyl ether extract. Moreover, the DPPH scavenging activity of the RC extract was equivalent to those of natural antioxidants (66%–78% at the concentrations of 0.05–0.1 mg.mL^−1^) acquired from fruits, and vegetables [23]. However, all assayed fractions exhibited inferior activities than that of the control (vitamin C), with 93.7%. The scavenging ability may act as a pronounced index, and Hintz et al. [5] described that the antioxidant activity of destined plant extracts have been coordinated to their reducing powers. The number of phenolic and flavonoid compounds have positive coordination with DPPH and ABTS radical scavenging activities because of hydrogen and electron contribution from hydroxyl groups of these compounds. In general, ABTS assay is considered as potentially more efficient than DPPH assay, since ABTS can estimate both hydrophilic and hydrophobic substances [24]. Among the seven extracts, RCC extract exhibited the magnificent antioxidant activities in both of the antioxidant assays; thus, GC-MS analysis was performed to acquire its component knowledge.

### 3.4. GC-MS Analysis

GC-MS is a productive technique in differentiation, recognition, and quantification of intricate mixtures (such as plant extraction) and confer the most sensitive recognition of bioactive compounds [25]. Thus, GC-MS was conducted to explore the volatile components of the RCC, which exhibited strong antioxidant and antimicrobial activity in RC extract. The GC-MS analysis of RCC resulted in the identification of seven compounds (Table 3) and chromatogram are presented in Figure S1. The analysis showing the existence of phenols, aldehydes, and several organosulfur compounds, in which 2-furancarboxyaldehyde was rarely reported in RCC.

The RCC extract showed major compounds, i.e., 2-furancarboxaldehyde, 5-methylfuran-2-carbaldehyde, and 2-methoxy-4-vinylphenol, which may be responsible for antimicrobial activity. According to the GC-MS results, the organosulfur compounds found in RCC extract have been shown to influence multiple biological effects, such as antioxidant, anti-inflammatory, and fungicides, as well as various pharmacological and therapeutic effects [1,5]. The spectrum of these sulfur-containing compounds relies substantially on the extraction methods of the RC, moreover, on the solvent conditions of processing [17].

The antimicrobial potential of the recognized commercially accessible compound was analyzed using the disc diffusion method as depicted in Table S3. Methylsulfonylmethane, 5-methylfuran-2-carbaldehyde, 2 purinol, and 4H-Pyran-4-one were not effective at all towards the tested microorganisms, even at higher concentration with 1 mg.mL^−1^. The 2-methoxy-4-vinylphenol and benzofuran exhibited the strong antibacterial activity and 2-Furancarboxaldehyde exhibited weak antibacterial activity; however, all compounds were not effective towards the tested fungal strains. The antibacterial activity of 2-methoxy-4-vinylphenol and benzofuran were less sensitive towards the tested microorganisms as compared to RCC extract at lower concentration of 0.5 mg.mL^−1^; however, at a higher concentration of 1 mg.mL^−1^, it exhibited comparable antibacterial activity, in terms of zone of inhibition (Table S3). 

### 3.5. In Silico Molecular Docking Mechanism

**Figure 2:**
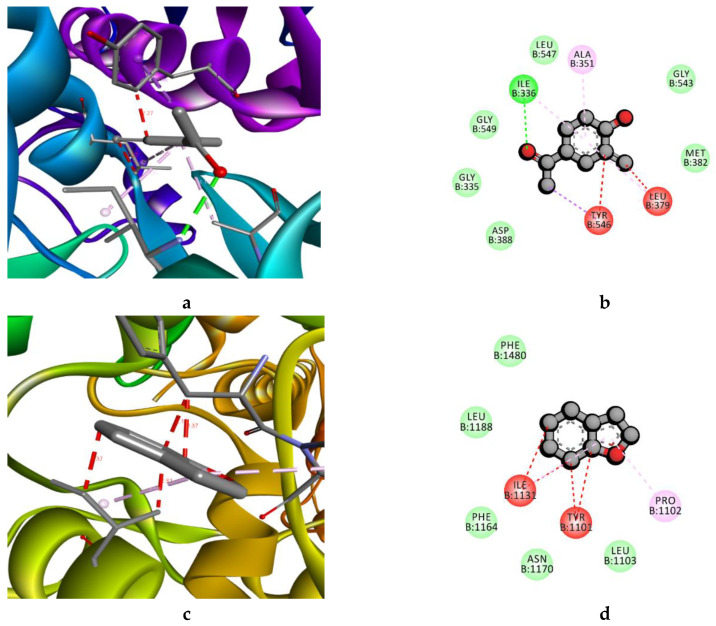
Docking analysis; (**a**,**b**). Three-dimensional (3D) and two-dimensional (2D) structures of the interactions between 2-Methoxy-4-vinylphenol and LpxC, respectively, and (**c**,**d**). The 3D and 2D structures of the interactions between benzofuran and 4PLB, respectively.

The molecular interactions results indicated that among the tested compounds, benzofuran and 2-Methoxy-4-vinylphenol showed the higher negative docking score against 4PLB (−8.229 Kcal.mol^−1^) and LpxC (−8.63 Kcal.mol^−1^), respectively (Table 3). LpxC, an emerging target in Gram-negative bacteria, leads to the synthesis of Lipid A biosynthetic pathway, which is an essential component for the survival of bacteria [9]. In addition, it makes it resistant to commonly used antibiotics [26]. The NBTI is another emerging target, which mediates breakdown of double-stranded DNA and participates in DNA replication and decatenation reactions [27]. In addition, NBTIs have proven to be resistant against fluoroquinolone antibiotics [28]. Therefore, the Benzofuran and 4PLB established the strong interactions with the hydrophobic side chain aliphatic (Ile1131), aromatic (Tyr1101), and unique amino acids Pro 1102 (Figure 2c,d). In case of the 2-Methoxy-4-vinylphenol, LpxC established interaction with the conventional hydrogen bond hydrophobic aliphatic side chain (Ile 336, Ala351, Leu379) and aromatic side chain Tyr546 (Figure 2a,b). The identified compounds have been previously reported in a number of studies as depicted in Table 3. The 2-methoxy-4-vinylphenol have been previously reported in a number of studies due to their wide range of biological activities, such as antimicrobial, antioxidant, anti-inflammatory, analgesic, and anti-germination [29]. With respect to benzofuran, benzofuran and its derivatives are well known for their biological activities, and have potential as emerging natural antimicrobial agents in the food industry [29]. In addition, 2-Furancarboxaldehyde has been identified in a number of studies; however, antimicrobial activity is not pronounced [30]. However, other identified compounds have also been reported in a number of studies due to their antioxidant and anti-inflammatory activity, not well-established for their antimicrobial activity [30,31]. Based on their antimicrobial activity, and previously reported studies, we conclude that 2-methoxy-4-vinylphenol are potential compounds for the antimicrobial activity of RCC extract. Further, we analyze the potential of RCC extract in food (beef) as an antimicrobial agent to enhance the shelf life of meat and meat products.

**Figure 3:**
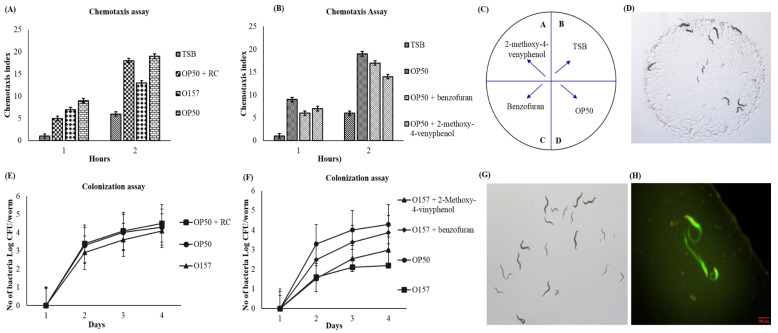
(**A**) Chemotaxis assay of RC extract compared between *E. coli* 0157 and *E. coli* OP50 (basic food for worms), (**B**) chemotaxis assay compared between OP50 and commercially available identified compounds; 2-methoxy-4-vinyphenol and benzofuran combined with OP50, respectively. (**C**) The model chart of chemotaxis assay. (**D**) Chemotaxis assay plate indicates the from the center of the plate worm move towards the test sample equally when compared with control (OP50). (**E**) Colonization assay indicates the durability of the work when fed [Pathogen—*E. coli* O157, and Plant extract (RCC extract)]. (**F**) Colonization assay indicates the durability of the work when fed [Post treatment of 2-methoxy-4-vinyphenol against pathogen—*E. coli* O157 + benzofuran against pathogen—*E. coli* O157 + 2,3 and *E. coli* 0157 (pathogen)]. (**G**) Stereomicroscopic image on the Morphology of *C. elegans* after the colonization assay. (**H**) The stereomicroscopic image indicates the green spots (Syto-9) healthy live cells of the *C. elegans* after the colonization assay.

Additionally, we perform cytotoxicity for its safety aspects, to determine the feasibility of developing an effective and safe antimicrobial agent for a natural preservative. The *C. elegans* model was used to determine the cytotoxic effect to avoid possible unwanted biological side effects, such as chemotaxis assay (Figure 3 and Table 4).

### 3.6. Effect of RCC on C. Elegans (In Vivo) Model against E. coli O157:H7

The effect of RCC extract on *C. elegans* chemotaxis assay results exhibited in Figure 3. The *C. elegans* fed with OP50 showed the highest chemotaxis index, which demonstrates that most of the worms attracted towards OP50 and RCC extract, combined with OP50, showed the comparable chemotaxis index. Less attraction of the worms for the RCC extract, combined with OP50, could be attributed to the benzene carboxylic acid group, which is one of the secondary metabolites present in *Brassica* vegetables [5]. Among the identified compounds, benzofuran and 2-methoxy-4-vinyphenol combined with OP50 showed a comparable chemotaxis index to OP50. As we discussed in Section 3.4 and Section 3.5, benzofuran and 2-methoxy-4-vinyphenol are potential compounds responsible for the antimicrobial activity of RCC extract.

The results of the colonization assay depicted in Figure 3E,F. The L4 stage of the worms failed to lay eggs compared to L1, L3, or L4 stages of worms when fed with OP50 (4.3 log CFU/5 worm), despite the fact that RCC extract combined with OP50 also acts as a substantial food source for worms, and similar results observed by commercially available identified compounds (Figure 3F). However, the results suggest that RCC extract exhibited protective and longevity effects on worms by reducing the *E. coli* O157:H7 population in the gut of the worm fed with *E. coli* O157:H7. This beneficial effect of RCC extract may be due to the presence of secondary plant metabolites [5], while worms fed with *E. coli* O157:H7 leads to an increase in colonization (4.2 log CFU/5 worm).

These results strongly suggest that RC extract contains promising phytochemical compounds that might be beneficial as a natural antimicrobial agent. Remarkably, the effect of RCC extract has not been previously confirmed with the *C. elegans* model system. 

### 3.7. Shelf Life Study

#### 3.7.1. Changes in Microbial Profile

**Figure 4:**
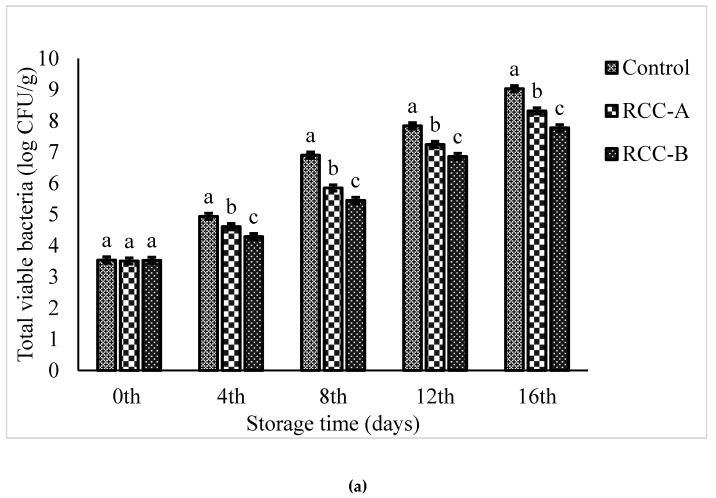

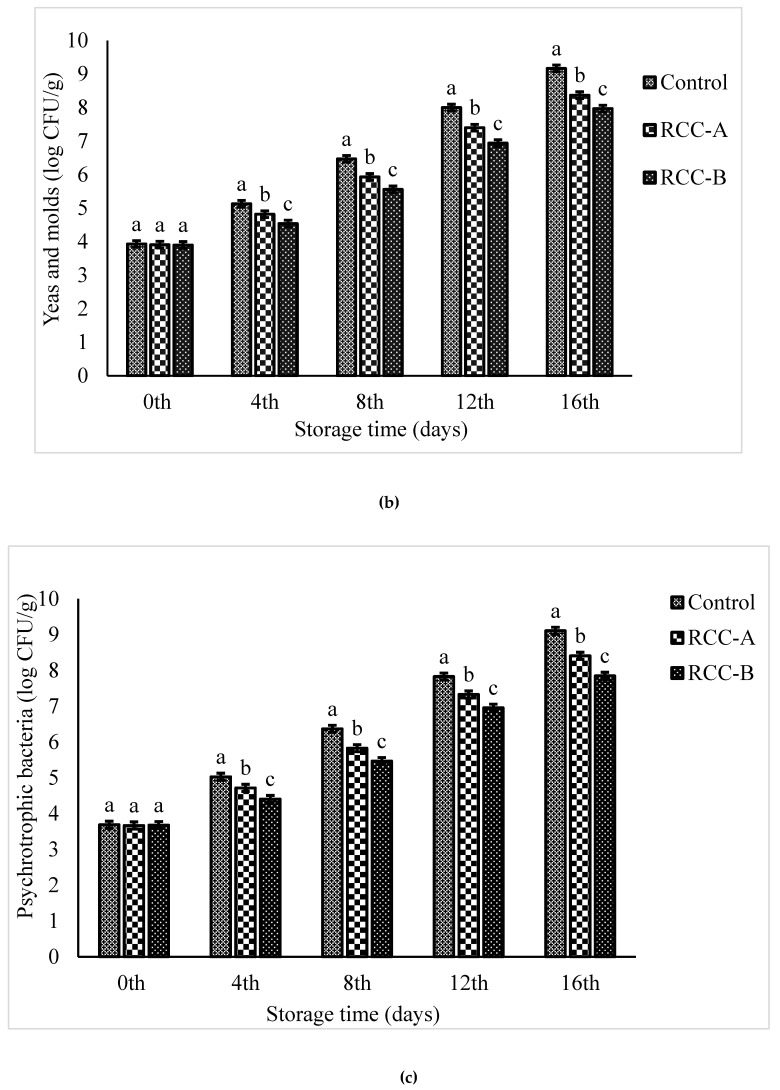
Effect of white cabbage extract A and B on (**a**) total viable count, (**b**) yeast and molds, and (**c**) psychrotrophic bacteria in beef meat during storage at 4 °C. Values representing mean ± SD of three replicates; different letters indicate significant difference (*p* < 0.05) for each time; same letters indicate no significant difference (*p* > 0.05) for each time.

Changes in TVC, psychrotrophic and yeast, and molds of beef samples are shown in Figure 4a–c. The results exhibit that the microbial population decreased with the addition of the natural extract, and showed longer storage. Among the experimental categories, control sample showed the swift increase in the number of microorganisms, followed by the samples treated with RCC-B and RCC-A. The addition of RCC extract derived in depletion in growth rate of TVC. The TVC was, initially, approximately 3.54, 3.51, and 3.53 CFU.g^−1^ for control, RCC-A and RCC-B, respectively (Figure 4a), which is consistent with the results of the fresh beef treated with chitosan [12]. By the end of the fourth day, the TVC of RCC-B treatment was significantly lower (*p* < 0.05) as compared to RCC-A and the control. TVC increased with respect to increase in storage period, and increased up to 6.9 log CFU.g^−1^ at day 8 for control, and at day 12 for RCC-A, which was evaluated as higher acceptability for fresh meat [32].

Psychrotrophic bacteria can comprise an important fraction of the natural microflora of beef products, which may cause spoilage of meat at 4 °C storage [32]. The initial psychrotrophic bacterial counts were found to be 3.68 log CFU.g^−1^ meat for all categories (*p* < 0.05). At the end of storage period, the highest psychrotrophic bacterial count was obtained for control (9.11 log CFU.g^−1^), followed by RCC-A (8.41 log CFU.g^−1^) and RCC-B (7.85 log CFU.g^−1^) (Figure 4b).

Yeast and molds were found to be another important part of natural microflora of beef, which are responsible for the spoilage of meat [32]. The initial yeast and mold counts were found to be 3.93, 3.91, and 3.90 log CFU.g^−1^ for control, RCC-A and RCC-B categories, respectively. Yeast and mold counts increased with the storage period, and reached to 9.17 log CFU.g^−1^ for control, followed by RCC-A and RCC-B with 8.37 and 7.97 log CFU.g^−1^, respectively, at the end of storage period (Figure 4c). Taken together, the pronounced increase in the count of all groups of microorganisms was noted in the C sample, substantially for TVC and psychrotrophic bacteria.

Natural plant extracts exhibited antimicrobial activity towards all test microorganisms. Several other studies suggest that shelf life of meat and meat products can be extended by compounds from plant extracts and essential oils or a combination of both [1,33,34,35]. To the best of our knowledge, the impact of RCC extracts on the microbiological stability of meat and meat products had not been investigated priorly. Fernández et al. [36] had denoted that 5% citrus extract treatment entirely restricted the growth of lactic acid bacteria (LAB) in beef meat balls during a 12-day storage period. In another study, lamb meat treated with rosemary extracts (10% and 20%) had lower extent of TVC during 21 days of storage in modified atmosphere packaging (70% O_2_, 30% CO_2_); however, no substantially significant differences were recorded among the variants [37]. Therefore, the microbial counts of all analyzed microorganisms were lower in beef samples treated with RCC extract; they could be used for delaying microbial growth and extending the shelf life of meat and meat products.

#### 3.7.2. Changes in pH

The effect of RCC extract on the pH of beef at 4 °C storage is depicted in Table 5. The initial pH of the C samples was 5.64 ± 0.02, which is consistent with reports by other authors [12]. During storage, the pH values of C and extract treated samples depleted slightly on the fourth day, and then gradually increased by the end of storage (Table 5); our results are consistent with the previous results [38]. Such a rise in the pH of control samples indicate the degree of meat spoilage, because of microbial or protein breakdown for the elevation of free amino acids guided to the generation of NH_3_, amines, and compounds of alkaline reaction, which ultimately cause an increase in pH [39]. During storage period, control samples displayed a significantly higher pH value than the treated samples, which is compatible with reports by other authors [12,38]. The pH of RCC-A and RCC-B were sustained throughout the storage period and no big change was perceived, which may be due to the presence of phenolic compounds (2-Methoxy-4-vinyphenol, 2-Purinol, and methysulfonylmethane). In addition, these compounds have previously been reported from other plant sources for their strong antioxidant activity [31,40].

#### 3.7.3. Changes in Moisture

The moisture content is a recurring parameter in food storage and preservation, and fresh meat typically contains higher moisture content (approximately 70%–75%). Alteration in moisture content may induce significant variations in the stability and quality of food. The lowest value of the moisture displayed the low sensitivity of meat to microbial growth. Overall, moisture content, cautiously, moderately depleted in all of the meat samples, including both C and RCC treated samples at 4 °C storage (Table 5). Although, the decrease in moisture content (%) was significantly lower (*p* < 0.05) in RCC-B treated samples when compared to control and RCC-A extract. Generally, irrespective of any type of meat, moisture content gradually decreased at 4 °C storage.

#### 3.7.4. Changes in TBARS

The effect of RCC polyphenolic extract on lipid oxidation (described as TBARS) at 4 °C storage is depicted in Table 5. The RCC extract influenced a substantial inhibitory effect on the production of TBARS, however, with diverse intensity. At the beginning of storage (day 0), no significant differences were distinguished among treatments and control. The TBARS values for all treated samples varied between 0.25 ± 0.05 to 0.51 ± 0.03 mg MDA.kg^−1^ meat showing a very low degree of lipid oxidation, however, gradually increased by the end of storage period. After 16 days of storage, TBARS values of RCC-A and RCC-B categories were 2.05 and 1.62 mg MDA.kg^−1^, respectively, where the control sample was obtained as 2.73 mg MDA.kg^−1^; our results are consistent with previously reported studies [32]. The RCC leafy extracts significantly (*p* < 0.05) depleted TBARS production compared to the C samples exhibiting an antioxidant effect. The lowest increase of TBARS values were observed in meat samples treated with RCC-B (2%).

Phytochemical compounds are renowned as effective antioxidants [1]. Hence, it was logical to anticipate that the utilization of natural plant extracts should restrict lipid oxidation and extend the shelf life of meat. The results of current studies identify the potential of RC extract as an effective natural antioxidant to extricate towards lipid oxidation under storage. The utilization of natural plant extracts affect the microbial population in meat products by restricting metabolic activity [5]. In previous studies, it has been reported that TBARS values, equivalent to or greater than 2 mg MDA.kg^−1^ meat, incorporate the threshold for determining off-odors and ‘off-taste’ for consumers [39]. Such high TBARS values were not documented in the current study. The values of TBARS in RCC-B treatment were considerably lower than in C and RCC-A treated samples (*p* < 0.05), indicating that the RCC-B treatment constructively protected from lipid oxidation of raw meat.

#### 3.7.5. Color Analysis

Color generation and stability were considered as salient markers of the quality of raw meat and meat products. Table 6 depicts the lightness (L*), redness (a*), and yellowness (b*) of meat with and without RCC extract at 4 °C storage. The highest a* values under storage were perceived for the C samples. The red color of a meat product is a salient factor aspired by the majority of consumers. If the redness values extend from 4.60 to 10.80, the product is recognized as brown [41]. Unfortunately, the usage of plant extracts did not permit to attain the desired red color. During the first 8 days of storage, only RCC-B treated meat samples were red. The a* values were calculated as 20.12 and 10.63, respectively. After 8 days, the a* value of the RCC-B sample was substantially higher than that of RCC-A treated samples, but still less than in control samples.

The b* values of all the samples increased in a consistent manner with the storage time. After 8 days of storage, the b* values of RCC-A and RCC-B treated samples increased significantly when compared to C samples. The yellowness of the meat was, thus, perhaps because of the color of the plant extracts. With respect to lightness, a subsequent decrease was observed for all meat samples; however, it remained stable in RCC-B treated samples when compared to control and RCC-A samples.

The results of the current study demonstrate that RCC extracts do not have a strong impact on color changes (L*, a*, b* values) in meat during storage. It had been described that red cabbage extracts, rich in anthocyanins, improve the red color of beef. In addition, [42] noted a decrease in color parameters, attributed to lipid oxidation, which may induce color changes in meat during storage. Although, in the current study, TBARS values were significantly lower for RCC-A and RCC-B meat samples as compared to the C samples. Moreover, the inclusion of natural plant extracts may instigate changes in the color parameters due to their own characteristic color.

#### 3.7.6. Textural Analysis

Texture plays a crucial role in the value of meat in terms of appearance. Hardness or tenderness has been observed as the most important attribute among texture characteristics to satisfy the consumer’s preference. Hardness or tenderness of meat can be defined as the force required to obtain a given distortion or a perforation in a product [43]. Therefore, to evaluate the meat quality, hardness was performed with other textural characteristics, such as cohesiveness, gumminess, springiness, and chewiness. Table 7 depicts the texture analysis. All texture characteristics undergo some changes during storage. Generally, all treatments and control samples showed no significant differences (*p* < 0.05) for texture characteristics throughout the storage period. With respect to effect of RCC extract, the RCC-B treated samples depicted a lower hardness, gumminess, and chewiness than the RCC-A and control samples after he fourth and eighth day of storage (*p* < 0.05). In general, hardness and chewiness are positively correlated; with the decrease of hardness, chewiness also decreased by the end of storage period. Cohesiveness and springiness were also lower in RCC-B as compared to RCC-A and C samples. According to Lund et al. [44], meat hardness could be ascribed to a higher influence in protein oxidation reactions, leading to the generation of crosslinking and polymerization in proteins [44].

In summary, our results demonstrate the effectiveness of RCC extract in inhibiting microbial growth, reducing lipid oxidation, maintaining organoleptic properties, and extending the shelf life of raw beef meat during storage at 4 °C for 16 days. Current trends of food preservation, considering consumer preference, lead to a depletion in the use of synthetic preservatives for natural additives. In addition, natural antimicrobial compounds from plant sources, such as RC, could effectively deplete the number of microorganisms tested in this study. Therefore, RCC extract application in the development of novel functional healthy meat products may be highly valuable and desirable in the meat industry. However, the use of natural extracts in meat products could be restricted because the addition of high amounts can adversely affect the organoleptic properties of the product. Hence, further studies are required to investigate the antimicrobial effects of concentration below sensorial thresholds and the acceptable level of such natural extracts in the meat industry.

## Figures and Tables

**Table 1:** Antimicrobial activity of different extracts of red cabbage (RC).

List of Microorganisms	Zone of Inhibition Diameter (mm)						
	CE	TE	DE	EEE	EtE	ME	DWE
**Gram-negative bacteria**							
*Salmonella. enterica typhimurium* ATCC 14,028	12.00 ± 0.01^b^	12.00 ± 0.04^b^	10.00 ± 0.02^a^	-	11.00 ± 0.03^ab^	10.00 ± 0.05^a^	-
*Escherichia. coli* ATCC 35,150	13.00 ± 0.02^c^	11.00 ± 0.03^b^	10.00 ± 0.05^ab^	-	10.00 ± 0.05^ab^	09.00 ± 0.06^a^	-
*Escherichia. coli* ATCC 43,894	11.00 ± 0.03^b^	10.00 ± 0.02^ab^	10.00 ± 0.04^ab^	-	11.00 ± 0.03^b^	09.00 ± 0.06^a^	-
**Gram-positive bacteria**							
*Staphylococcus. aureus* ATCC 13,150	14.00 ± 0.04^bc^	11.00 ± 0.02^ab^	13.00 ± 0.04^b^	-	-	10.00 ± 0.05^a^	-
*Staphylococcus. aureus* ATCC 12,600	11.00 ± 0.02^ab^	11.00 ± 0.05^ab^	10.00 ± 0.06^a^	-	11.00 ± 0.07^ab^	-	-
*Listeria. monocytogenes* ATCC 19,118	12.00 ± 0.02^b^	12.00 ± 0.05^b^	10.00 ± 0.05^a^	-	13.00 ± 0.02^c^	-	-
*Bacillus. cereus* ATCC 14,579	14.00 ± 0.03^b^	12.00 ± 0.05^ab^	11.00 ± 0.06^a^	-	11.00 ± 0.02^a^	-	-
**Fungi**							
*Candida. albicans* KCTC 7965	15.00 ± 0.01^b^	-	13.00 ± 0.05^a^	14.00 ± 0.03^ab^	-	-	-
*Aspergillus. fumigatus* KCTC 6145	08.50 ± 0.02^a^	-	-	10.00 ± 0.05^b^	-	-	-
*Aspergillus. flavus var. flavus* KCTC 6143	10.00 ± 0.05^ab^	10.00 ± 0.03^ab^	-	09.00 ± 0.03^a^	-	-	-
*Aspergillus. niger* KCTC 6317	09.00 ± 0.02^a^	09.00 ± 0.01^a^	10.00 ± 0.03^ab^	09.00 ± 0.05^a^	-	-	-

CE: Chloroform extract, TE: Toluene Extract, DE: Dichloromethane Extract, EEE: Ethyl Ether Extract, EtE: Ethanol Extract, ME: Methanol Extract, DWE: Distilled Water Extract. -: no inhibition zone, a–c letters are according to increasing mean values, different letters in each row for each extract’s antimicrobial activity represent statistically significant difference (*p* < 0.05), same letters in each row for each extract’s antimicrobial activity represents non-significant difference (*p* > 0.05).

**Table 2:** Thermostability of the chloroform extract of RC.

List of Microorganisms	Heating at 95 °C for Different Times (min); Zone of Inhibition (mm)		
	5	45	90
**Gram-negative bacteria**			
*S. enterica typhimurium* ATCC 14,028	10.00 ± 0.02^a^	13.00 ± 0.01^b^	15.00 ± 0.03^c^
*E. coli* ATCC 35,150	09.00 ± 0.01^a^	12.00 ± 0.02^b^	09.00 ± 0.01^a^
*E. coli* ATCC 43,894	10.00 ± 0.02^a^	10.00 ± 0.02^a^	12.00 ± 0.03^b^
**Gram-positive bacteria**			
*S. aureus* ATCC 13,150	10.00 ± 0.02^a^	14.50 ± 0.01^b^	15.50 ± 0.03^bc^
*S. aureus* ATCC 12,600	10.00 ± 0.02^a^	12.00 ± 0.02^b^	12.00 ± 0.03^b^
*L. monocytogenes* ATCC 19,118	11.00 ± 0.03^a^	15.00 ± 0.02^b^	16.30 ± 0.05 ^c^
*B. cereus* ATCC 14,579	12.00 ± 0.04^a^	15.00 ± 0.02^b^	20.00 ± 0.03^c^
**Fungi**			
*C. albicans* KCTC 7965	08.00 ± 0.02^a^	10.50 ± 0.03^b^	14.00 ± 0.01^c^
*A. fumigatus* KCTC 6145	08.50 ± 0.01^a^	11.00 ± 0.05^b^	11.00 ± 0.03^b^
*A. flavus var. flavus* KCTC 6143	10.00 ± 0.03^a^	11.30 ± 0.02^b^	13.00 ± 0.01^c^
*A. niger* KCTC 6317	09.00 ± 0.01^a^	10.00 ± 0.04^ab^	12.30 ± 0.03^c^

-: no inhibition zone, a–c letters are according to increasing mean values, different letters in each row for each extract’s antimicrobial activity represent statistically significant difference (*p* < 0.05), same letters in each row for each extract’s antimicrobial activity represents non-significant difference (*p* > 0.05).

**Table 3:** Docking results of identified compounds from RCC extract.

Compound	Chemical Formula	Area (%)	Molecular Weight (g.mol^−1^)	Docking Score (Kcal.mol^−1^)		Activity	References
				4PLB	LpxC		
Methylsulfonylmethane	C_2_H_6_O_2_S	0.04	94.133	−6.10	−6.32	Antioxidant, anti-inflammatory, anti-cancer	[26]
2-Furancarboxaldehyde	C_5_H_4_O_2_	0.11	96.084	−5.84	−5.70	Antibacterial	[27,28]
5-Methylfuran-2-carbaldehyde	C_6_H_6_O_2_	0.05	110.111	−6.94	−6.96	Pharmaceutical properties, organic inhibitor	[29]
4H-Pyran-4-one	C_5_H_4_O_2_	0.06	96.084	−5.40	−5.91	Pharmacological activity,	[28]
Benzofuran	C_8_H_6_O	0.01	118.133	−8.229	−8.11	Anti-inflammatory analgesic, antimicrobial	[30]
2-Purinol	C_5_H_4_N_4_O	0.04	136.111	−6.14	−6.04	Antioxidant, potential of novel pharmaceuticals; anti-proliferative	[31]
2-Methoxy-4-vinyphenol	C_9_H_10_O_2_	0.01	150.174	−7.70	−8.63	Antimicrobial, antioxidant, anti-inflammatory, analgesic, anti-germination	[30]

**Table 4:** Cytotoxic properties of different extracts of RC in MCF-7 cell line.

Sr. No	Plant Extract	IC_50_ (µg.mL^−1^) MCF-7
1	RC-Chloroform Extract	>50
2	RC-Dichloromethane Extract	>50
3	RC-Toluene Extract	>50
4	RC-Ethyl ether Extract	>50
5	RC-Ethanol Extract	>50
6	RC-Methanol Extract	>50
7	Tamoxifen	10.08

IC: Half-maximal inhibitory concentration, MCF: Michigan Cancer Foundation-7 (breast cancer cell line).

**Table 5:** The effect of RCC extract on the pH, thiobarbituric acid reactive substances (TBARS), and moisture values of beef during storage at 4 °C.

Quality Attributes	Storage Time (days) at 4 °C					
	Treatments	0	4	8	12	16
pH	Control	5.64 ± 0.02^a^	5.37 ± 0.01^a^	6.29 ± 0.01^a,b^	6.61 ± 0.01^b^	6.89 ± 0.03^b^
	RCC-A	5.63 ± 0.01^a^	5.31 ± 0.05^a^	5.69 ± 0.05^a^	5.61 ± 0.05^a^	5.73 ± 0.03^a^
	RCC-B	5.61 ± 0.01^a^	5.23 ± 0.08^a^	5.51 ± 0.08^a^	5.53 ± 0.01^a^	5.62 ± 0.05^a^
TBARS (mg MDA/kg)	Control	0.29 ± 0.01^a^	0.99 ± 0.01^a,b^	1.45 ± 0.01^a,b^	1.84 ± 0.08^a^	2.73 ± 0.04^b^
	RCC-A	0.25 ± 0.05^a^	0.55 ± 0.01^a^	0.98 ± 0.01^a^	1.51 ± 0.01^a^	2.05 ± 0.03^a^
	RCC-B	0.26 ± 0.06^a^	0.46 ± 0.09^a^	0.89 ± 0.01^a^	1.42 ± 0.08^a^	1.62 ± 0.05^a^
Moisture (%)	Control	43.28 ± 0.03^a^	41.07 ± 0.02^b^	38.40 ± 0.02^a^	36.05 ± 0.06^a,b^	32.16 ± 0.08^a^
	RCC-A	43.17 ± 0.05^a^	40.79 ± 0.01^a^	39.14 ± 0.02^a,b^	35.57 ± 0.07^a^	32.33 ± 0.05^a^
	RCC-B	43.31 ± 0.06^a^	41.06 ± 0.04 ^b^	40.05 ± 0.03^b^	37.21 ± 0.05^b^	34.04 ± 0.07 ^b^

All values are expressed as mean ± SD of three replicates; a and b letters are according to increasing mean values; different letters in each column for each quality analysis represent statistically significant difference (*p* < 0.05); same letters in each column for each quality analysis represents non-significant difference (*p* > 0.05).

**Table 6:** Changes in color parameters of beef meat treated with RC extract during storage at 4 °C.

Treatments	Parameters	Storage Time (days) at 4 °C				
		0	4	8	12	16
Control	L*	48.44 ± 0.08^a^	47.42 ± 0.02^a^	44.12 ± 0.08^a^	40.96 ± 0.06^a^	36.72 ± 0.06^a^
RCC-A		48.31 ± 0.05^a^	47.00 ± 0.03^a^	44.96 ± 0.04^ab^	41.82 ± 0.09^b^	37.66 ± 0.06^b^
RCC-B		48.26 ± 0.02^a^	47.47 ± 0.08^a^	45.54 ± 0.05^b^	42.79 ± 0.03^c^	39.99 ± 0.05^c^
Control	a*	15.41 ± 0.05^a^	11.82 ± 0.02^ab^	11.44 ± 0.02^c^	10.33 ± 0.01^c^	6.13 ± 0.03^c^
RCC-A		15.65 ± 0.04^a^	12.78 ± 0.03^b^	7.45 ± 0.03^a^	4.46 ± 0.02^a^	2.93 ± 0.08^a^
RCC-B		15.20 ± 0.04^a^	11.12 ± 0.04^a^	8.63 ± 0.07^b^	6.42 ± 0.02^b^	4.76 ± 0.04^b^
Control	b*	6.48 ± 0.04^a^	4.14 ± 0.05^a^	2.43 ± 0.02^a^	1.31 ± 0.08^a^	-0.43 ± 0.05^a^
RCC-A		6.75 ± 0.04^a^	5.54 ± 0.08^b^	5.45 ± 0.07^b^	2.81 ± 0.01^b^	0.58 ± 0.06^b^
RCC-B		6.61 ± 0.02^a^	5.89 ± 0.02^b^	5.92± 0.03^b^	4.69 ± 0.06^c^	2.56 ± 0.02^c^

All the values are mean ± SD, L*: lightness, a*: redness, b*: yellowness, letters a–c are given according to the increasing mean values; values for each parameter sharing the same letter in each column represents non significance difference at *p* > 0.05; values for each parameter sharing different letter in each column represents significance difference at *p* < 0.05.

**Table 7:** Texture profile analysis of RC extract on muscle of beef meat during storage at 4 °C. All the values are mean ± SD; letters **a**—**c** are given according to the increasing mean values; values for each parameter sharing same letter in each column represents non significance difference at *p* > 0.05; values for each parameter sharing different letter in each column represents significance difference at *p* < 0.05.

Days	Treatments	Texture parameters				
		Hardness (g)	Cohesiveness	Springiness (mm)	Chewiness (mJ)	Gumminess (g)
0th	Control	1160 ± 0.02^a^	0.53 ± 0.03^a^	1.61 ± 0.01^a^	10.9 ± 0.05^a^	583 ± 0.03^a^
	RCC-A	1190 ± 0.04^a^	0.53 ± 0.01^a^	1.53 ± 0.03^a^	10.9 ± 0.06^a^	590 ± 0.04^a^
	RCC-B	1185 ± 0.05^a^	0.56 ± 0.03^a^	1.42 ± 0.03^a^	10.4 ± 0.04^a^	559 ± 0.05^a^
4th	Control	810 ± 0.04^a^	0.74 ± 0.02^a^	1.78 ± 0.02^a^	10.5 ± 0.03^a^	599 ± 0.01^a^
	RCC-A	905 ± 0.01^b^	0.53 ± 0.03^a^	1.69 ± 0.04^a^	12.00 ± 0.02^b^	618 ± 0.03^a^
	RCC-B	1000 ± 0.03^c^	0.53 ± 0.03^a^	1.73 ± 0.05^a^	10.8 ± 0.02^a^	636 ± 0.03^a^
8th	Control	710 ± 0.03^a^	0.56 ± 0.05^a^	1.27 ± 0.01^a^	6.30 ± 0.03^a^	406 ± 0.05^a^
	RCC-A	805 ± 0.05^b^	0.53 ± 0.04^a^	1.13 ± 0.03^a^	11.8 ± 0.05^b^	561 ± 0.06^b^
	RCC-B	855 ± 0.04^b^	0.59 ± 0.05^a^	1.48 ± 0.02^a^	5.20 ± 0.03^b^	557 ± 0.07^b^
12th	Control	500 ± 0.06^a^	0.97 ± 0.03^a^	0.81 ± 0.04^a^	5.00 ± 0.01^b^	380 ± 0.03^a^
	RCC-A	600 ± 0.03^b^	0.74 ± 0.01^a^	1.45 ± 0.03^a^	9.40 ± 0.04^b^	400 ± 0.03^a^
	RCC-B	720 ± 0.04^c^	0.56 ± 0.03^a^	0.57 ± 0.05^a^	4.10 ± 0.03^a^	463 ± 0.02^ab^
16th	Control	395 ± 0.05^a^	0.59 ± 0.02^a^	2.93 ± 0.03^a^	10.0 ± 0.01^a^	248 ±0.04^a^
	RCC-A	420 ± 0.06^a^	0.16 ± 0.06^a^	2.26 ± 0.01^a^	10.2 ± 0.06^a^	260 ±0.05^a^
	RCC-B	530 ± 0.03^b^	0.32 ± 0.02^a^	2.78 ± 0.05^a^	18.8 ± 0.00^b^	389 ± 0.03^b^

## Supplementary Materials

The following are available online at https://www.mdpi.com/2304-8158/9/5/568/s1, Figure S1: GC-MS chromatogram of RCC extract, Table S1: Quantitative phytochemical analysis of different extracts of RC, Table S2: Antimicrobial activity of the compounds present in RC.
