# Supplementary material for: Bioactive Potential of 2-Methoxy-4-vinylphenol and Benzofuran from Brassica oleracea L. var. capitate f, rubra (Red Cabbage) on Oxidative and Microbiological Stability of Beef Meat
Source: Foods. 2020 May 4;9(5):568. doi: 10.3390/foods9050568 (PMC7278745; doi:10.3390/foods9050568)
Supplement: Supplementary file 1 [file foods-09-00568-s001.pdf]

Table S1. Quantitative phytochemical analysis of different extracts of RC

Sr. No	Phytocompound	CE	DE	EEE	TE	EtE	ME	DWE
1	Phenol	+	+	+	+	-	+	-
2	Saponin	+	+	-	-	-	-	-
3	Glycosides	+	+	+	+	-	-	-
4	Steroids	-	-	+	+	-	-	-
5	Terpenoids	-	-	-	-	-	-	-
6	Tanins	-	-	-	-	-	-	-
7	Flavonoid	+	+	+	+	-	-	-

-: not present, +: present, CE: Chloroform Extract, TE: Toluene Extract, DE: Dichloromethane Extract, EEE: Ethyl Ether Extract, EtE: Ethanol Extract, ME: Methanol Extract, DWE: Distilled Water Extract

Table S2: Antimicrobial activity of the compounds present in RC

List of microorganisms	Zone of inhibition (mm)					
	2-Methoxy-4-vinyphenol		Benzofuran		5-Methylfuran-2-carbaldehyde	
	**0.5 mg.mL^{-1}**	**1.0 mg.mL^{-1}**	**0.5 mg.mL^{-1}**	**1.0 mg.mL^{-1}**	**0.5 mg.mL^{-1}**	**1.0 mg.mL^{-1}**
**Gram-negative bacteria**						
ATCC 14028	09.00 ± 0.03^{b}	15.00 ± 0.01^{a}	09.00 ± 0.03^{b}	11.00 ± 0.05^{a}	-	-
ATCC 35150	10.00 ± 0.02^{b}	15.00 ± 0.04^{b}	-	-	-	-
ATCC 43894	11.00 ± 0.03^{b}	20.00 ± 0.05^{b}	-	-	-	-
**Gram-positive bacteria**						
ATCC 13150	11.00 ± 0.01^{a}	15.00 ± 0.05^{a}	-	-	-	-
ATCC 12600	10.00 ± 0.04^{a}	17.00 ± 0.03^{a}	11.00 ± 0.01^{a}	11.00 ± 0.04^{a}	-	-
ATCC 19118	10.00 ± 0.02^{a}	19.00 ± 0.03^{b}	10.00 ± 0.04^{a}	10.00 ± 0.03^{a}	-	-
ATCC 14579	10.00 ± 0.05^{a}	20.00 ± 0.05^{a}			-	-
**Fungi**						
KCTC 7965	11.00 ± 0.01^{a}	11.00 ± 0.05^{a}	-	-	-	-
KCTC 6145	10.00 ± 0.04^{a}	13.00 ± 0.05^{a}	-	-	-	-
KCTC 6143	10.00 ± 0.04^{a}	13.00± 0.03^{a}	-	-	-	-
KCTC 6317	10.00 ± 0.03^{a}	11.00 ± 0.05^{a}	-	-	-	-

List of microorganisms	Zone of inhibition (mm)					
	2-Purinol		Methylsulfonylmethane		2-Furancarboxaldehyde	
	**0.5 mg.mL^{-1}**	**1.0 mg.mL^{-1}**	**0.5 mg.mL^{-1}**	**1.0 mg.mL^{-1}**	**0.5 mg.mL^{-1}**	**1.0 mg.mL^{-1}**
**Gram-negative bacteria**						
ATCC 14028	-	-	-	-	-	-
ATCC 35150	-	-	-	-	-	-
ATCC 43894	-	-	-	-	-	-
**Gram-positive bacteria**						
ATCC 13150	-	-	-	-	-	-
ATCC 12600	-	-	-	-	-	-
ATCC 19118	-	-	-	-	-	-
ATCC 14579	-	-	-	-	-	-
**Fungi**						
KCTC 7965	-	-	-	-	-	-
KCTC 6145	-	-	-	-	-	-
KCTC 6143	-	-	-	-	-	-
KCTC 6317	-	-	-	-	-	-

Figure S1. GC-MS chromatogram of RCC extract

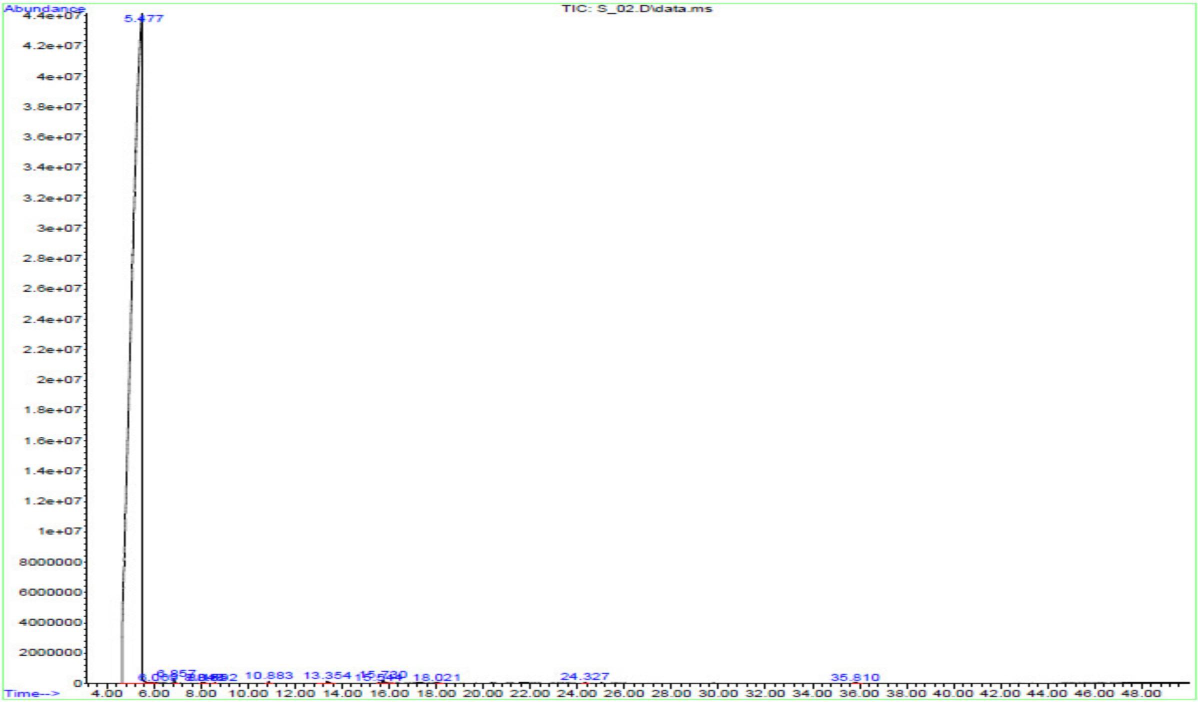

Peak	R.T time	Area
1	5.479	1.340E + 10
2	6.859	7101224
3	13.355	8058714
4	15.545	1660089
5	15.732	14721382
6	18.020	1747328
7	35.812	4990492

Library report - C:\Database\W8N05ST.L

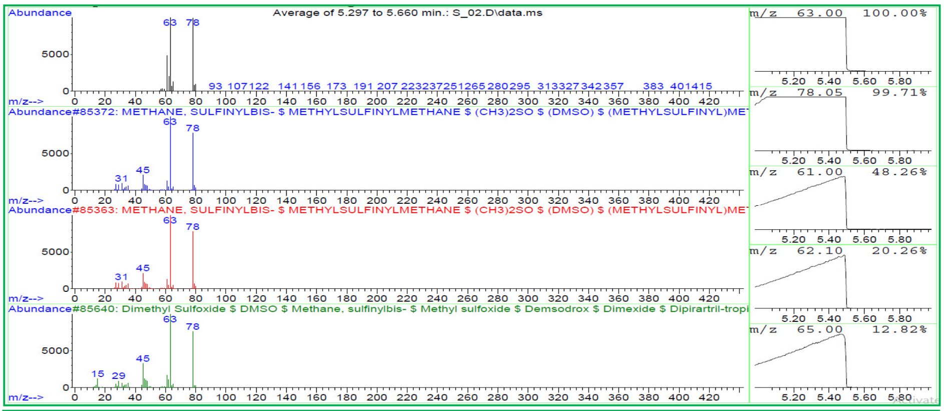

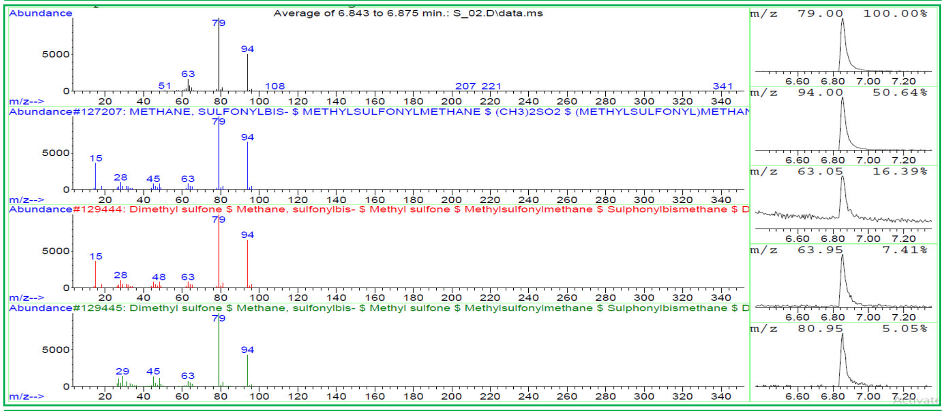

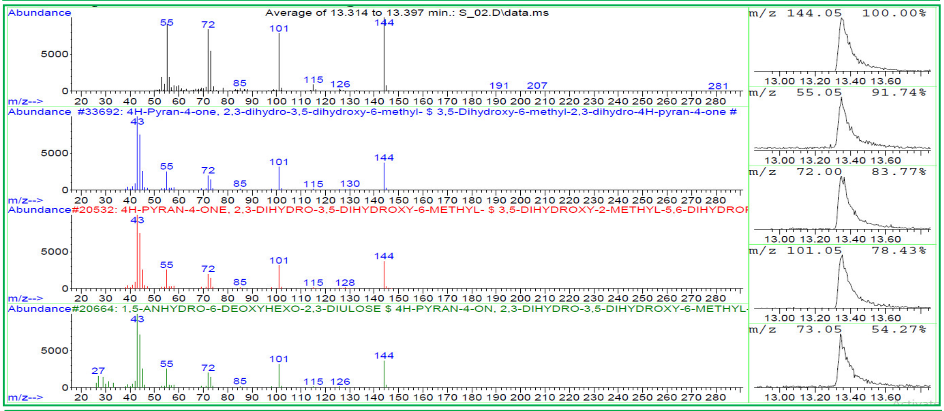

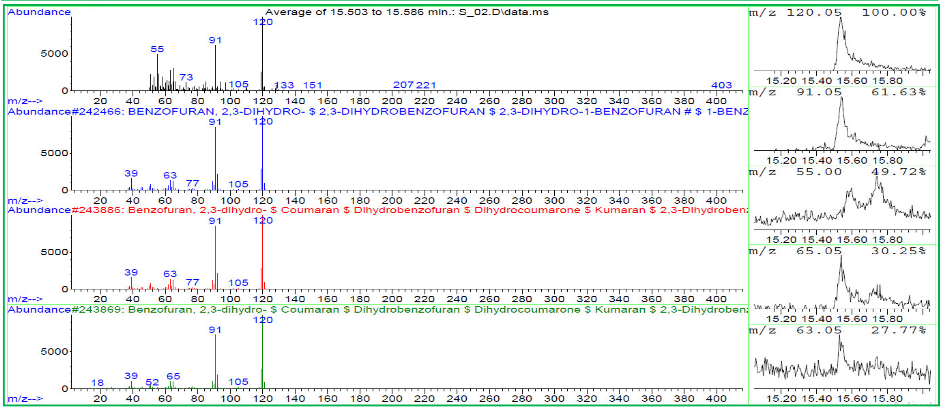

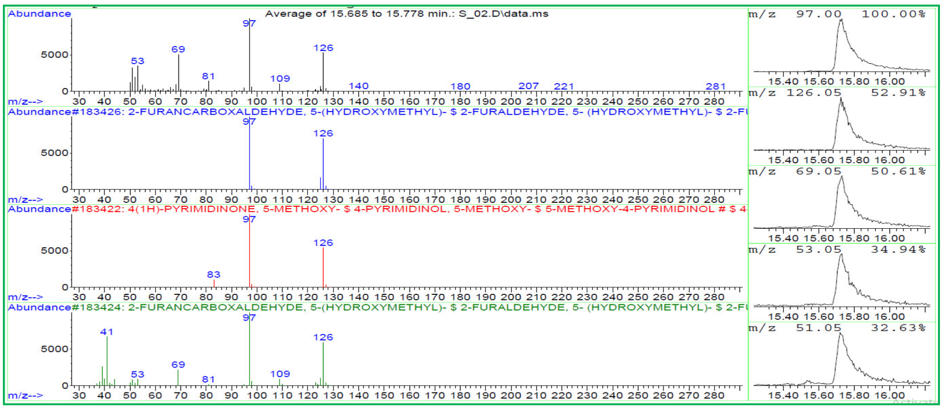
Average of 15.685 to 15.778 min.: S_02.D\data.ms
#183426: 2-FURANCARBOXALDEHYDE, 5-(HYDROXYMETHYL)- $ 2-FURALDEHYDE, 5- (HYDROXYMETHYL)- $ 2-F
#183422: 4(1H)-PYRIMIDINONE, 5-METHOXY- $ 4-PYRIMIDINOL, 5-METHOXY- $ 5-METHOXY-4-PYRIMIDINOL # $ 4
#183424: 2-FURANCARBOXALDEHYDE, 5-(HYDROXYMETHYL)- $ 2-FURALDEHYDE, 5- (HYDROXYMETHYL)- $ 2-F
Abundance
m/z-->
m/z 97.00 100.00%
m/z 126.05 52.91%
m/z 69.05 50.61%
m/z 53.05 34.94%
m/z 51.05 32.63%
